# Physical and Emotional Interventions in Modulating Neuroplasticity: A Narrative Review of Recent Evidence

**DOI:** 10.7759/cureus.98446

**Published:** 2025-12-04

**Authors:** Britty Babu, Fathima Mohideen Bawa, Gauri Parvathy, Anya R Gupta, Gurnoor S Gill, Alfredo A Paredes, Adja Seitaj, Daniela Herbert, Manish Gupta

**Affiliations:** 1 Department of Medicine, Tbilisi State Medical University, Tbilisi, GEO; 2 Department of Global Health, Ghent University, Ghent, BEL; 3 Department of Science, Florida Atlantic University High School, Boca Raton, USA; 4 Department of Medicine, Florida Atlantic University Charles E. Schmidt College of Medicine, Boca Raton, USA; 5 Department of Technology and Clinical Trials, Advanced Research, Deerfield Beach, USA

**Keywords:** neural plasticity, neurologic care, neurologic disorder, neurology and critical care, neuroplastic adaptations

## Abstract

Neuroplasticity, the brain’s capacity to adapt structurally and functionally in response to experiences, is a key factor in recovery from neurological conditions. Growing interest has focused on physical and emotional interventions as potential modulators of neuroplastic processes. A narrative review was conducted using a structured PubMed search of articles published between January 2018 and March 2023. Studies were included if they examined physical activity, emotional well-being, or cognitive training, with outcomes relevant to neuroplasticity. Publications outside these parameters, non-English studies, and case reports were excluded. Twenty-seven articles met the inclusion criteria.

The literature described a range of possible influences. Exercise was frequently linked with markers such as brain-derived neurotrophic factor (BDNF) and synaptic plasticity. Emotional interventions, including stress reduction and mindfulness, were reported to have variable effects on neural adaptation. Cognitive training was discussed in relation to functional recovery in conditions such as stroke and traumatic brain injury (TBI). Findings across studies were heterogeneous, and conclusions were limited by differences in study designs and outcome measures.

Current evidence suggests that both physical and emotional interventions may influence neuroplasticity, though results are not uniform. The diversity of methodologies and measured outcomes indicates that more rigorous and standardized research is needed. While the reviewed literature points to potential benefits of physical and emotional interventions in modulating neuroplasticity, evidence remains preliminary. Further high-quality longitudinal studies are required to clarify their role in neurological rehabilitation and clinical care.

## Introduction and background

Neuroplasticity, defined as the brain’s ability to reorganize and adapt in response to internal and external stimuli, has become a central focus in neuroscience and clinical medicine. This phenomenon encompasses processes such as synaptic remodeling, dendritic branching, and neurogenesis, which collectively support learning, memory, and recovery from injury [1-5]. In the context of neurological disorders, neuroplasticity offers an important framework for developing interventions that may enhance rehabilitation and functional outcomes [6].

Recent studies have explored how both physical and emotional factors may influence these adaptive mechanisms. Physical activity has been linked to improvements in cognitive function and age-related brain health, suggesting a role in mitigating cognitive decline [7,8]. At the same time, emerging literature highlights the interplay between stress, immune signaling, and neural circuits, underscoring the contribution of emotional states to brain function [8]. Non-pharmacological interventions, such as music therapy and structured physical activity, are also being studied in neurodegenerative conditions, including Alzheimer’s disease, as potential means of maintaining or restoring neural function [9,10].

Traditional approaches to neurological care remain essential, yet they may not fully utilize the brain’s capacity for adaptive change [10]. Integrating physical exercise, mindfulness, and nutrition has been proposed as a way to stimulate neuroplastic processes, potentially enhancing outcomes for patients with stroke, traumatic brain injury (TBI), and neurodegenerative diseases [11,12]. Furthermore, interventions targeting both physical and emotional well-being may have the dual benefit of improving quality of life and supporting neural adaptation in individuals living with chronic neurological conditions [13-15].

As highlighted in numerous medical studies and clinical research, including those published in prestigious journals such as "Stroke," "Neurobiology of Stress," and "Neurodegenerative Diseases," this understanding represents a pivotal step toward advancing neurological care [8]. Neurological conditions - including those addressed by music therapy - ranging from stroke and TBIs to neurodegenerative diseases like Alzheimer's, pose multifaceted challenges to patients and healthcare providers alike [9]. These conditions often lead to disruptions in neural pathways, resulting in cognitive and motor impairments that significantly diminish a patient's quality of life. Traditional treatment approaches, while valuable, may fall short in fully capitalizing on the brain's remarkable ability to adapt, or neuroplasticity [10]. It is within this context that we embark on a journey to explore the synergistic potential of physical exercise, mindfulness-based stress reduction, and nutrition-rich interventions in enhancing neuroplasticity [11]. These interventions, as documented in medical literature, can stimulate neural growth, synaptic plasticity, and cognitive recovery.

In pursuit of improving clinical outcomes and overall well-being for patients facing neurological challenges, it is essential to understand how physical and emotional interventions may meaningfully influence neuroplasticity. Rather than restating established mechanisms, this section focuses on the broader context in which neuroplasticity operates - specifically, how neurological disorders disrupt adaptive brain processes and why targeted interventions may restore or enhance them. Neurological conditions, such as stroke, TBI, and neurodegenerative diseases, alter functional neural networks, often resulting in lasting cognitive or motor impairments. Because the brain retains the capacity to reorganize and compensate for these deficits, interventions that strategically engage neural systems hold promise for improving recovery trajectories.

Within this framework, integrated physical and emotional approaches have emerged as potential tools for strengthening adaptive neural responses. These strategies - from structured exercise programs to stress-modulating practices - seek to support the brain’s intrinsic ability to reorganize, restore functional connectivity, and optimize learning and recovery processes. By situating neuroplasticity as a central therapeutic target rather than an abstract concept, these interventions highlight a shift toward rehabilitation models that leverage the brain’s biological adaptability [12]. As healthcare continues to evolve toward more personalized and mechanism-driven treatment paradigms, the potential for neuroplasticity-based interventions to enhance long-term outcomes becomes increasingly relevant. The promise of these approaches lies not only in their ability to complement traditional care, but also in their capacity to foster a more resilient neurological future for affected individuals. In this review, we explore how these integrated strategies may translate into tangible improvements in clinical practice and patient quality of life.

This review was guided by the following clinical question: In patients with neurological conditions, do integrated physical and emotional interventions, compared with standard care or no intervention, lead to enhanced neuroplasticity and improved clinical outcomes? The primary goal of this paper is to synthesize recent findings on how physical activity, stress management, mindfulness, and cognitive training may influence neuroplasticity in neurological populations. By highlighting both consistencies and gaps in the literature, this review aims to provide clinicians and researchers with a clearer understanding of the potential role of these interventions in supporting recovery and rehabilitation.

This study evaluated search strategies for finding high-quality studies on treatment and systematic reviews in PubMed, with a manual review of each article for each issue of 64 journal titles for the years 2018 to 2023. The study shows that 48 (11.5%) of review articles met the criteria for systematic reviews [15].

Search strategy

Relevant articles published from 2018 to 2023 were searched primarily in the PubMed database. The study explored how early-life environmental enrichment impacts cognitive performance and neuroplasticity in male and female rats, with potential implications for human health [16]. The following Medical Subject Headings (MeSH) terms and keywords were used in the PubMed database search: ("neuroplasticity" OR "brain plasticity" OR "neural plasticity") AND ("physical activity" OR "exercise" OR "physical training" OR "physical therapy" OR "motor learning" OR "sensory stimulation" OR "environmental enrichment" OR "neurogenesis" OR "cognitive training" OR "emotion" OR "emotional state" OR "stress" OR "neurotransmitters" OR "psychological factors" OR "mental health" OR "psychiatric disorders").

Research questions

This review systematically examined existing literature in order to address the following questions: What is the current state of knowledge regarding the impact of physical activity and exercise on neuroplasticity in human models [17]? How do different types of physical training, such as aerobic exercise, resistance training, and motor skill learning, influence neuroplasticity, and what are the underlying mechanisms [18]? What is the relationship between environmental enrichment, sensory stimulation, and neuroplastic changes in the brain, and how does this relate to cognitive function [19]? What role does neurogenesis play in the context of physical and emotional factors affecting neuroplasticity, and how can it be modulated [20]? How do emotional states - including stress, anxiety, and positive emotions - impact neuroplasticity, and what are the potential therapeutic implications for mental health and psychiatric disorders [21]? What neurotransmitters and neurobiological pathways mediate the effects of emotional states on neuroplasticity, and how can these pathways be targeted for intervention [22]? Are there gender- or age-related differences in the influence of physical and emotional factors on neuroplasticity, and, if so, what are the implications for personalized healthcare [23]?

Inclusion and exclusion criteria

Eligible articles consisted of published scientific research or reviews that focused specifically on the topic of physical and emotional influences on neuroplasticity. The review considered articles published between 2018 and 2023 to maintain relevance and currency [24]. Participants included individuals of diverse ages, backgrounds, and health statuses. Various research types, such as experimental, observational, review, meta-analysis, and clinical trial designs, were considered. Articles investigated the impact of physical and emotional factors on neuroplasticity, including variables such as physical activity, exercise, and emotional states (e.g., stress, anxiety, and positive emotions), as well as related interventions [25]. Relevant outcome measures encompassed neurobiological changes, cognitive alterations, and behavioral modifications associated with neuroplasticity [26].

Conversely, exclusion criteria guided the selection process. Articles falling outside the scope of primary research or reviews (e.g., case reports, books, and editorials) were excluded from consideration [27]. The review also adhered to strict temporal boundaries, excluding articles published before January 2018 or after March 2023. Articles that did not directly address the impact of physical and emotional factors on neuroplasticity, or that focused on unrelated topics, were not deemed eligible for inclusion. Studies conducted solely on non-human models, without clear relevance to human neuroplasticity, were also excluded [28]. Finally, to ensure uniformity and ease of analysis, articles published in languages other than English were excluded due to potential language limitations.

These inclusion and exclusion criteria collectively established a robust framework for selecting articles that contributed to a comprehensive understanding of the influence of physical and emotional factors on neuroplasticity, in the context of human subjects [29].

Study selection

Two independent reviewers, identified as MSSF and GCJS, selected articles for inclusion in the study based on a set of predetermined inclusion criteria. These criteria consisted of articles written and published between the years 2018 and 2023 in the PubMed database, encompassing studies that involved humans, utilized “physical exercise” or its related terms as an intervention, encompassed various brain tissues, and investigated neuroplasticity [30-32]. Articles were assessed against the PICOS criteria, which included the following aspects: Participants (humans), Interventions (physical exercise), Comparisons (PE vs. no PE groups), Outcomes (neuroplasticity components), and Study design (human studies), as shown in the flow chart, where 27 relevant data points were found [33-59]. Studies were included if they examined neuroplasticity in the context of physical, emotional, or lifestyle-based interventions; studies unrelated to these domains or lacking neuroplasticity outcomes were excluded. In the subsequent stage, a meticulous comparison was conducted between the search results and the evaluation of titles and abstracts to ensure alignment with the eligibility criteria. Selected abstracts were subjected to a comprehensive review by two additional independent researchers, and any discrepancies were resolved through consensus [32]. 

**Figure 1:**
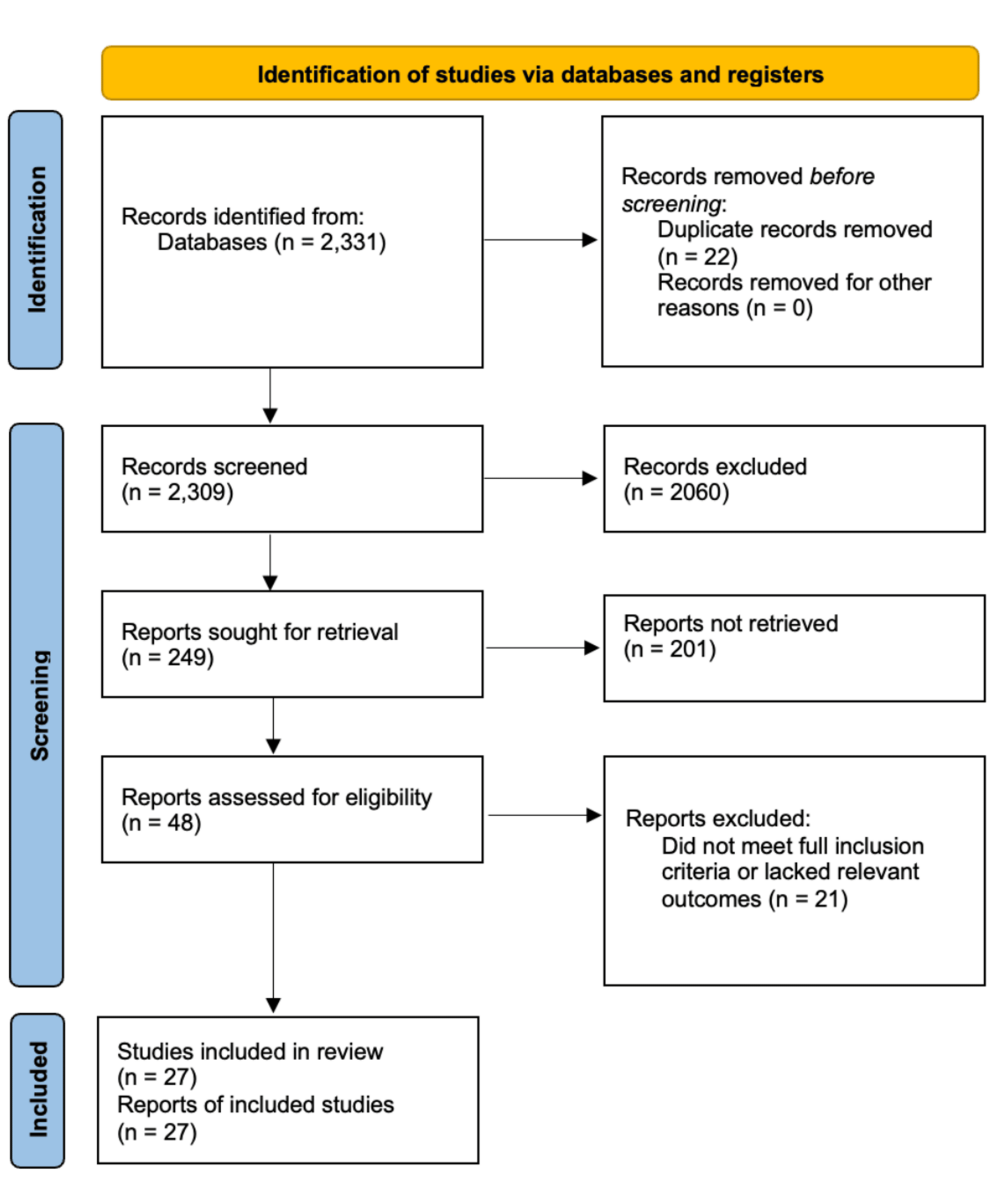
Flow Diagram of Article Selection Process

A total of 2,331 records were identified through the PubMed database. After removing 22 duplicate records, 2,309 unique records remained for screening. Following title and abstract review, 2,060 records were excluded for not meeting the inclusion criteria. The remaining 249 records were sought for full-text retrieval, of which 201 could not be retrieved or contained insufficient data for analysis. A total of 48 full-text articles were assessed for eligibility, and 21 were excluded for not meeting full inclusion criteria or lacking relevant neuroplasticity outcomes. The final N = 27 studies met all inclusion criteria and were included in the qualitative synthesis. A simplified screening flowchart is included for transparency in how studies were identified and screened; however, this review does not follow PRISMA methodology, and the diagram is not intended to represent a formal PRISMA flowchart (Figure 1).

Because this is a narrative review, no formal risk-of-bias assessment or structured data extraction was performed. Also, given the narrative nature of the review and the heterogeneity of study designs, a tabulated summary was not included. Given the substantial heterogeneity across study designs, populations, and neuroplasticity outcome measures (e.g., functional magnetic resonance imaging (fMRI), electroencephalogram (EEG), serum biomarkers, and behavioral tests), a formal meta-analysis was not appropriate. Therefore, findings are synthesized qualitatively.

## Review

Neuroplasticity and its role in recovery

Neuroplasticity, a fundamental neurobiological process, holds immense significance in neurological recovery, particularly in the context of conditions like stroke. This phenomenon encompasses structural and functional changes within the brain, involving mechanisms such as long-term potentiation and synaptic pruning [33]. However, despite strong mechanistic plausibility, much of the evidence originates from small cohorts or controlled experimental settings, which may limit broad clinical applicability.

For instance, in stroke rehabilitation, when a region of the brain is damaged due to reduced blood flow, adjacent areas can undergo neuroplastic changes to compensate for the lost function [34]. This adaptability allows patients to regain motor skills and cognitive functions, making neuroplasticity a cornerstone of post-stroke recovery. Carey et al. demonstrated, in a cohort of stroke patients, that structured motor retraining improved upper limb function by 25% and was associated with enhanced connectivity in the sensorimotor cortex [33]. Nevertheless, these improvements vary widely depending on stroke severity, timing of intervention, and individual recovery trajectories, which complicates direct comparisons across studies.

Mensen et al. further confirmed that sleep-mediated synaptic reorganization contributed to motor recovery, highlighting the importance of rest in post-stroke rehabilitation [34]. Xiong et al. reported that neural stem cell transplantation improved behavioral outcomes and increased brain-derived neurotrophic factor (BDNF) levels, highlighting the regenerative potential of neuroplasticity in TBI models [35]. Yet, stem-cell-based studies are predominantly preclinical, and their translation to human populations remains limited by ethical, methodological, and safety considerations.

Collectively, these studies reinforce the notion that structural and functional remodeling in the brain underlies recovery across various neurological pathologies. At the same time, the heterogeneity in study design, sample size, and outcome measures underscores the need for more standardized methodologies to strengthen the quality and comparability of evidence.

Moreover, in cases of TBI, the brain's ability to rewire itself plays a vital role in the recovery process. By engaging in targeted rehabilitation exercises and cognitive therapies, individuals with TBI can harness neuroplasticity to rebuild neural pathways, recover memory, and enhance cognitive function [35]. In neurodegenerative diseases such as Alzheimer's, where cognitive decline is a hallmark, interventions aimed at enhancing neuroplasticity - such as cognitive training and physical exercise - have shown promise in slowing disease progression and improving cognitive performance [36]. Overall, neuroplasticity offers a dynamic avenue for recovery in various neurological conditions, underscoring its essential role in the rehabilitation and treatment of patients with disorders affecting the nervous system, as shown in Table 1 [37-41].

**Table 1:** Key Physical and Emotional Factors Influencing Neuroplasticity and Their Mechanisms BDNF, brain-derived neurotrophic factor

Factor	Influences on neuroplasticity	Mechanism and implications	References
Physical activity	Increases synaptic plasticity	Synaptic plasticity refers to the ability of synapses (connections between neurons) to strengthen or weaken.	[38]
	Enhances dendritic branching	Increased synaptic plasticity supports learning and memory.	
	Augments neurogenesis	Enhanced dendritic branching allows neurons to form more connections, facilitating information processing.	
	Elevates BDNF levels		
	Increases cerebral blood flow		
Environmental enrichment	Stimulates neuroplastic changes	Environmental enrichment involves exposing individuals to complex and stimulating environments.	[39]
	Promotes cognitive function	This can lead to structural changes in the brain, supporting cognitive development and neuroplasticity.	
Emotional stress	Chronic stress can impair synaptic plasticity	Chronic stress can lead to dendritic atrophy (shrinkage) and reduced neurogenesis, affecting learning and memory.	[40]
	Stress can impact neurogenesis and cognitive function, reducing neurogenesis and affecting learning and memory	Acute stress, when controlled, may trigger adaptive acute stress, when controlled, may trigger adaptive neuroplasticity mechanisms that improve resilience and cognitive flexibility.	
	Controlled acute stress may stimulate adaptive changes		
Positive emotions	Positive emotions and mindfulness practices can enhance synaptic plasticity	Positive emotions can enhance the release of neurotrophic factors, which support the growth and maintenance of neurons and synapses.	[41]
	Improve neurogenesis	Mindfulness practices, such as meditation, have been associated with increased gray matter density and enhanced cognitive functions.	
	Enhance cognitive functioning		

Physical Interventions and Neuroplasticity 

These therapies, such as constraint-induced movement therapy and task-specific training, stimulate neuroplastic changes, promoting motor and functional improvements in conditions like stroke and spinal cord injuries [42]. However, the strength of evidence varies considerably across studies, with many relying on small sample sizes or short intervention periods, which limits the ability to generalize these findings to broader neurological populations [40-42].

Exercise and neuroplasticity: A growing body of research highlights the profound influence of exercise on neuroplasticity in neurological patients. Numerous studies have demonstrated that regular physical activity can lead to notable improvements in brain structure and function, particularly in conditions like stroke and Parkinson's disease [43]. These benefits include increased synaptic plasticity, enhanced dendritic branching, and augmented neurogenesis, which collectively contribute to cognitive and motor recovery. Mechanistically, exercise fosters neuroplastic changes by elevating BDNF levels, a key factor in neural growth and synaptic plasticity. Furthermore, aerobic exercise increases cerebral blood flow, promoting nutrient and oxygen delivery to the brain, further supporting neuroplasticity [44]. Wu et al. found that dance-based aerobic interventions in older adults with mild cognitive impairment led to significant improvements in memory and executive function, accompanied by magnetic resonance imaging (MRI) evidence of increased hippocampal gray matter [42]. El-Sayes et al. showed that 12 weeks of moderate aerobic exercise elevated serum BDNF levels and enhanced motor-evoked potentials, confirming cortical excitability changes [43]. Similarly, Kumar et al. demonstrated that sensorimotor rehabilitation improved oral-motor control and functional cortical reorganization, while Pronk linked diet-exercise synergy to cognitive improvement [44,45]. These results indicate that physical activity promotes measurable neuroplastic adaptations across brain regions. Still, the heterogeneity of exercise type, intensity, duration, and participant characteristics across studies introduces variability, which complicates direct comparisons and limits the ability to determine universally optimal exercise prescriptions for neuroplastic enhancement.

Nutrition and neuroplasticity: Recent research underscores the intricate interplay between nutrition and neuroplasticity, particularly in the context of neurological conditions. Emerging studies highlight the role of key nutrients, such as omega-3 fatty acids, antioxidants, and certain vitamins (e.g., B vitamins), in promoting synaptic plasticity, neurogenesis, and cognitive function [45]. Moreover, dietary patterns rich in antioxidants, polyphenols, and anti-inflammatory components - as observed in the Mediterranean diet - are linked to enhanced neuroplasticity. Understanding the impact of nutrition on brain plasticity is crucial, as it offers promising avenues for interventions in neurological disorders, emphasizing the potential therapeutic value of dietary approaches in optimizing brain health and function [46]. Pronk observed that individuals following a Mediterranean diet, rich in omega-3 fatty acids and polyphenols, exhibited higher hippocampal volume and improved working memory performance [45]. Maharjan et al. supported this by showing that lifestyle interventions combining diet and physical activity enhanced neurogenesis markers in older adults [20]. These findings underscore that nutrition exerts a measurable effect on brain morphology and function, complementing the neurobiological benefits of exercise. However, many nutritional studies rely on observational designs rather than randomized controlled trials, making it difficult to establish causality and increasing the possibility that unmeasured lifestyle factors contribute to the observed neuroplastic benefits.

Sleep and neuroplasticity*: *During sleep, particularly in the deeper stages, like slow-wave sleep, the brain engages in critical functions such as synaptic pruning, memory consolidation, and the removal of waste products. Adequate sleep quality and duration are essential for optimizing these neuroplastic processes [47]. In neurological patients, disrupted sleep patterns can hinder neuroplasticity, potentially impeding recovery. Therefore, prioritizing sleep hygiene and addressing sleep disorders can be integral components of therapeutic strategies aimed at enhancing neuroplasticity and neurological outcomes [48]. Salehinejad et al. demonstrated that sleep-dependent synaptic upscaling improves learning retention and task performance the following day [46]. Pickersgill et al. also found that optimizing sleep quality, when combined with exercise and diet, enhances neuroplastic capacity by supporting synaptic recovery and energy metabolism [47]. These studies affirm that sleep serves as a critical modulator of neuroplastic adaptation. Still, differences in how sleep quality is measured, along with individual variability in sleep architecture, limit the ability to standardize findings across studies or determine the most effective sleep-related interventions for enhancing neuroplasticity.

Emotional Interventions and Neuroplasticity

Stress and neuroplasticity:* *Stress elicits complex effects on neuroplasticity in neurological patients. Chronic stress can detrimentally impact synaptic plasticity, neurogenesis, and cognitive function, which are prevalent in conditions like PTSD and major depressive disorder [49]. Conversely, controlled acute stress responses may stimulate adaptive neuroplasticity mechanisms, enhancing cognitive resilience. To mitigate stress-related neuroplasticity impairments, therapeutic interventions like mindfulness-based stress reduction and cognitive-behavioral therapy, along with pharmacological approaches, are explored to modulate stress responses and support neurological recovery [50]. Ozbeyli et al. revealed that pharmacological modulation of stress pathways mitigated hippocampal neuronal loss and improved behavioral resilience in stress models [49]. Van Dam et al. and Madu emphasized that mindfulness-based interventions improved attentional control and emotional regulation through increased prefrontal activation and decreased amygdala reactivity [23,50]. Crăciun added that positive emotional training enhanced cognitive flexibility and functional connectivity in aging populations [22]. Together, these findings illustrate that emotional regulation directly modulates brain plasticity mechanisms. However, stress-related neuroplasticity research often relies on heterogeneous intervention models, making it difficult to compare outcomes or determine which stress-modulating strategies provide the most durable neural benefits.

Positive emotions and neuroplasticity: Research indicates that positive emotions and mindfulness practices can exert a beneficial influence on neuroplasticity in patients with neurological conditions. Studies have shown that cultivating positive emotional states and engaging in mindfulness techniques can enhance synaptic plasticity, neurogenesis, and cognitive functioning, offering potential therapeutic benefits [51]. Mechanistically, these effects may be attributed to the release of neurotrophic factors, reduced stress-related neuroinflammation, and increased connectivity within brain networks associated with emotional well-being, collectively contributing to improved neuroplasticity in individuals with neurological disorders [52]. Nonetheless, many studies examining positive emotions and mindfulness depend on self-reported measures and short-term follow-up, which may overestimate sustained neuroplastic changes or fail to capture long-term behavioral impacts.

Comparative Studies and Clinical Outcomes 

Comparative studies assessing the effectiveness of physical and emotional interventions in neurological patients, compared to standard treatments or no intervention, have provided valuable insights. These investigations often reveal significant improvements in neuroplasticity markers, such as increased BDNF levels and enhanced synaptic plasticity, associated with physical exercise and emotional well-being interventions. Additionally, cognitive function demonstrates substantial enhancements in these comparative studies, highlighting potential clinical benefits [53,54]. Kim et al. compared physical rehabilitation alone versus combined physical and mindfulness interventions, finding that the integrated group achieved higher functional gains and elevated cortical gray matter density on fMRI [53]. DeMaster et al. similarly demonstrated enhanced neurobehavioral outcomes in preterm infants receiving environmental enrichment and sensory stimulation [51]. Price and Duman concluded that emotional and cognitive training collectively improved depressive symptom scores and neuroplastic biomarkers, reinforcing the synergistic potential of multidomain interventions [54]. These findings emphasize the importance of incorporating holistic approaches, encompassing both physical and emotional well-being, into neurological care. Such strategies hold promise for optimizing neuroplasticity and ultimately improving outcomes. Still, the comparative evidence remains limited by small sample sizes, differing intervention intensities, and inconsistent outcome measures, making it challenging to determine whether multidomain programs outperform single-modality interventions across all neurological populations.

Discussion

The findings from the reviewed studies collectively highlight the complementary roles of physical and emotional interventions in modulating neuroplasticity. Exercise interventions, including aerobic activity, resistance training, and dance-based programs, consistently demonstrated structural and functional brain adaptations. These changes were supported by increased levels of BDNF, improved cerebral perfusion, and enhanced synaptic efficiency [33-36,42-45]. Emotional and mindfulness-based interventions, on the other hand, were associated with strengthened prefrontal-limbic connectivity, reduced stress reactivity, and greater emotional regulation [22,23,49,50]. Together, these findings emphasize that targeted behavioral interventions can meaningfully alter neural circuits and contribute to improved cognition, mood, and functional recovery.

When comparing modalities, studies that combined physical and emotional interventions yielded the greatest neuroplastic benefits [51-58]. For example, Kim et al. and Wu et al. reported that integrated exercise and mindfulness programs produced larger gains in cortical gray-matter density and cognitive flexibility than single-domain interventions [42,53]. These synergistic effects likely reflect the recruitment of multiple neurobiological pathways, with exercise enhancing peripheral neurotrophin signaling and mindfulness optimizing stress-related circuitry. Such multimodal approaches highlight the translational potential of lifestyle-based therapies in clinical neurorehabilitation and mental-health prevention.

Future research directions

Despite encouraging outcomes, several methodological gaps remain. Considerable heterogeneity in study designs, participant demographics, and neuroplasticity markers (e.g., fMRI, EEG, and serum BDNF) limits direct comparisons and meta-analytic synthesis. Many investigations relied on short intervention periods or small sample sizes, restricting generalizability. Standardization of assessment protocols, and the inclusion of long-term follow-up measures, are critical for determining the durability of neuroplastic gains.

One compelling avenue for future research to address these gaps is the in-depth exploration of the intricate mechanisms underlying the interaction between physical and emotional factors, and neuroplasticity, in neurological patients. While existing studies have established a link, the precise molecular and cellular processes driving this relationship remain a subject of ongoing exploration. Researchers can employ advanced neuroimaging techniques, such as fMRI and positron emission tomography (PET), to observe real-time changes in neural networks during integrated interventions. Additionally, molecular biology approaches, including gene expression analysis and proteomics, can provide insights into the specific neurobiological pathways involved. By elucidating these mechanisms, future studies can offer a more comprehensive understanding of how physical and emotional interventions induce neuroplastic changes.

The concept of personalized medicine has gained traction in recent years, and its application to neurological conditions presents an exciting avenue for research. Future studies can focus on identifying biomarkers that predict individual responses to integrated interventions. By conducting genetic profiling and analyzing the neurobiological profiles of patients, researchers can tailor treatment plans to maximize neuroplasticity for each individual. This approach holds the potential to optimize outcomes, as interventions can be customized based on an individual's unique genetic and neurobiological makeup. Moreover, the integration of artificial intelligence and machine learning algorithms can assist in developing predictive models that guide treatment decisions, ushering in an era of precision neurorehabilitation.

To fully appreciate the long-term effects of integrated physical and emotional interventions on neuroplasticity, future research should prioritize longitudinal studies. Such investigations would involve tracking patients over extended periods, ideally spanning several years. Longitudinal studies can assess the durability of neuroplastic changes induced by integrated interventions, and their impact on patients' clinical outcomes and quality of life. These studies should encompass diverse neurological conditions, including neurodegenerative diseases, to examine the potential for interventions to slow disease progression. Methodologically, researchers can employ a combination of neuroimaging, cognitive assessments, and patient-reported outcomes to comprehensively evaluate the sustained benefits of integrated approaches.

The future of neuroplasticity research in neurological patients necessitates cross-disciplinary collaboration. Researchers from neuroscience, psychology, exercise physiology, nutrition, and other relevant fields should come together to design comprehensive studies that account for the multifaceted nature of neurological conditions. Collaborative efforts can yield innovative interventions that combine physical exercises, emotional well-being strategies, dietary modifications, and cognitive training, ensuring a holistic approach to neurorehabilitation. Across the 27 studies reviewed, consistent patterns emerged - interventions combining physical and emotional modalities produced superior neuroplastic and functional outcomes compared with single-domain approaches. Most trials demonstrated improvements in BDNF, cortical volume, or connectivity indices. However, heterogeneity in design and measurement scales underscores the need for standardized assessment protocols to better compare outcomes across populations and modalities. Additionally, cross-disciplinary research teams can leverage a diverse set of expertise to address complex questions, such as gender- and age-related differences in the influence of physical and emotional factors on neuroplasticity [59].

As research in neuroplasticity advances, it is imperative to address ethical considerations and ensure informed consent in studies involving vulnerable populations, such as patients with severe neurological conditions. Ethical guidelines should be established to safeguard the rights and well-being of research participants. Researchers must transparently communicate the potential risks and benefits of interventions, especially when experimental treatments are involved. Moreover, research should prioritize inclusivity and diversity to ensure that findings are applicable to a wide range of individuals, accounting for differences in age, gender, socioeconomic status, and cultural background.

Integral to the future of neuroplasticity research in neurological patients is the assessment of cost-effectiveness. While integrated interventions hold promise, it is essential to evaluate their economic implications. Researchers can conduct rigorous cost-benefit analyses to determine the financial feasibility of implementing such interventions in healthcare settings. This includes considering the cost of interventions, potential reductions in healthcare expenses due to improved outcomes, and the overall return on investment. Policymakers, healthcare providers, and insurance companies can utilize this information to make informed decisions about integrating these interventions into standard care protocols.

## Conclusions

The evidence presented supports the notion that integrated physical and emotional interventions can enhance neuroplasticity, leading to improved outcomes in patients with neurological conditions. By capitalizing on the brain's remarkable adaptability, these interventions offer a holistic approach to neurorehabilitation. Tailoring treatment plans that combine physical exercises, emotional well-being strategies, and cognitive training can expedite recovery trajectories, reduce healthcare costs, and enhance patients' overall quality of life. While the research reviewed primarily focuses on specific interventions, the collective body of evidence underscores the potential for integrated approaches to optimize neuroplasticity. As healthcare providers and researchers continue to explore this dynamic field, the future holds promise for innovative interventions that harness the full potential of neuroplasticity in neurological care, offering hope and improved outcomes to countless individuals facing these challenging conditions.
